# Strigolactones in Plant Responses to Salt Stress: Regulatory Mechanisms and Application Potential

**DOI:** 10.3390/plants15132052

**Published:** 2026-07-02

**Authors:** Tangnaer Jieensi, Qiuping Fu, Linfeng Hu, Jian Huang, Tong Qi

**Affiliations:** 1 Institute of Agricultural Resources and Environment, Xinjiang Academy of Agricultural Sciences, Urumqi 830091, China; tangnar0605@163.com (T.J.); hulinfeng@xaas.ac.cn (L.H.); 2 College of Water Conservancy and Civil Engineering, Xinjiang Agricultural University, Urumqi 830052, China; qiupingfu@xjau.edu.cn

**Keywords:** strigolactones, salt stress, stress adaptation, physiological responses, exogenous application

## Abstract

Salt stress severely restricts plant growth and reduces crop yield. Strigolactones (SLs) are carotenoid-derived phytohormones involved in the regulation of plant salt tolerance. Salt stress can modulate the expression of SL biosynthetic and signaling genes, thereby affecting SL accumulation and signaling responses. SLs also interact with abscisic acid (ABA), reactive oxygen species (ROS), and other signaling molecules to coordinate downstream stress responses. At the physiological level, SLs alleviate salt stress by maintaining Na^+^/K^+^ homeostasis, enhancing osmotic adjustment and antioxidant defense, and reducing damage to the photosynthetic system. In addition, SLs can enhance plant resource acquisition and adaptive capacity under salt stress by regulating root architecture and promoting hyphal branching of arbuscular mycorrhizal fungi (AMF). This review focuses on SL-mediated regulation of plant salt tolerance at the molecular and physiological levels and further summarizes exogenous SL application strategies for alleviating salt stress, as well as research progress on key genes in the SL pathway for the genetic improvement of salt tolerance. Clarifying the potential of SLs in regulating plant responses to salt stress could provide new insights into sustainable crop production in saline-alkali environments.

## 1. Introduction

Soil salinization is an increasing constraint on agricultural production worldwide. According to the 2024 soil report by the Food and Agriculture Organization of the United Nations (FAO), salinity affects over 1.381 billion hectares of land globally, representing approximately 10.7% of the world’s land area [1]. In addition, salinization affects approximately one-third of irrigated farmland worldwide [2]. This poses substantial risks to agricultural sustainability and food security. Salt stress disturbs physiological and biochemical homeostasis and impairs plant growth and development, which eventually compromises crop yield and quality [3]. Therefore, improving crop salt tolerance and developing effective strategies are of great significance for maintaining stable crop productivity under saline conditions.

To cope with salt stress, plants activate complex regulatory networks, with phytohormones serving as key signals that coordinate growth, development, and stress adaptation [4]. Strigolactones (SLs) are carotenoid-derived terpenoid lactone phytohormones [5]. In recent years, SLs have attracted increasing attention for their regulatory roles in plant growth, development, and adaptation to environmental stresses. Growing evidence indicates that SLs positively regulate plant responses to salt stress [6]. Therefore, clarifying the potential links between SLs and related signaling pathways, together with the associated physiological and biochemical mechanisms under salt stress, will deepen our understanding of the regulatory networks governing plant salt tolerance.

SLs were first identified as chemical signals that stimulate the germination of root-parasitic plant seeds [7]. Subsequently, they were found to induce hyphal branching of arbuscular mycorrhizal fungi (AMF), thereby promoting the establishment of plant–AMF symbiosis [8]. Later studies established SLs as endogenous plant hormones that regulate multiple aspects of plant growth and development [9]. Among the known functions of SLs, the inhibition of axillary bud outgrowth and shoot branching is the most widely characterized. In addition, SLs contribute to root architecture remodeling, including primary and lateral root development and root hair formation, as well as leaf senescence and reproductive development [10]. Beyond developmental regulation, SLs also help alleviate stress damage and improve plant adaptation to adverse environments [11].

Salt stress can cause multiple forms of damage, including osmotic stress, ion toxicity, and secondary oxidative damage [12,13]. Accumulating evidence indicates that SLs regulate plant responses to salt stress at both molecular and physiological levels. SLs can help maintain physiological homeostasis by alleviating oxidative damage, enhancing water retention capacity and membrane stability, and improving ion homeostasis and osmotic adjustment [14]. In addition, SLs enhance plant salt tolerance by optimizing root architecture and promoting AMF symbiosis, thereby improving plant water and nutrient uptake efficiency [15].

SLs are important factors in plant responses to salt stress and show broad application prospects in the cultivation of salt-tolerant crops and the development of novel stress-mitigation regulators. At present, the molecular regulatory mechanisms, physiological responses, and application strategies of SLs under salt stress have not been systematically integrated. Therefore, this review aims to summarize the mechanisms by which SLs participate in plant responses to salt stress, assess their potential value in improving crop stress tolerance, and establish an overall framework for SL involvement in salt stress responses. Meanwhile, this review identifies key knowledge gaps in current research and proposes future research directions, providing a theoretical basis for addressing salt stress challenges and improving agricultural productivity.

## 2. SL Biosynthesis and Signaling

SL biosynthesis begins with all-trans-β-carotene, which is converted into carlactone (CL), a central precursor of SLs, through isomerization by the carotenoid isomerase DWARF27 (D27) and sequential cleavage by carotenoid cleavage dioxygenases CCD7 and CCD8. These early steps are highly conserved across studied plant species [16]. Biosynthetic steps downstream of CL formation are more diverse and largely account for SL structural diversity [17]. CL is then exported from plastids and further oxidized by cytochrome P450 monooxygenase MAX1 to form carlactonoic acid (CLA) [18]. In *Arabidopsis thaliana*, CLA is methylated by carlactonoic acid methyltransferase (CLAMT) to form methyl carlactonoate (MeCLA), which is subsequently hydroxylated by LATERAL BRANCHING OXIDOREDUCTASE (LBO) to generate SL-related compounds [19].

Recent studies have revealed that the species specificity of SL biosynthesis is mainly reflected in downstream modification steps. During these steps, members of the CYP722C family play key roles in the structural diversification of canonical SLs, and their catalytic products vary among species. In tomato (*Solanum lycopersicum*) and cowpea (*Vigna unguiculata*), CYP722C enzymes mediate the conversion of CLA into orobanchol-type SLs [20], whereas CYP722C in cotton (*Gossypium arboreum*) converts CLA into 5-deoxystrigol (5DS), a representative strigol-type SL [21]. In addition, P450 enzymes such as CYP706C and CYP728B are also involved in downstream structural modification of SLs. In maize (*Zea mays*), ZmCYP706C37 participates in the conversion of CL/CLA-derived intermediates and promotes the formation of non-canonical SLs, including zealactone, zealactol, and zealactonoic acid [22]. In rice (*Oryza sativa*), OsCYP706C2 participates in the CL-derived non-canonical SL biosynthetic branch and promotes the formation of 4-oxo-methyl carlactonoate (4-oxo-MeCLA) [23]. In sorghum (*Sorghum bicolor*), CYP728B35 enzyme further hydroxylates 5DS to form sorgomol [24]. These findings indicate that P450 enzyme-mediated downstream modification is a key driver of SL structural diversity.

Although SLs show structural diversity, SL signal transduction is relatively conserved across different plant species. In the canonical SL signaling pathway, D14, an α/β-hydrolase superfamily protein, functions as the SL receptor. Ligand perception induces conformational changes in D14 and promotes its association with the F-box protein MAX2 in the SCF complex. The resulting receptor complex targets SMXL/D53 transcriptional repressors for ubiquitination-mediated degradation, thereby derepressing downstream target gene expression [25,26].

Recent studies have further expanded our understanding of SL signaling mechanisms. SL-induced D14 degradation can still occur in the absence of MAX2 and SMXLs, suggesting that alternative E3 ubiquitin ligases or proteasome-independent degradation pathways may be involved. This process may function as a negative feedback mechanism that limits the duration and intensity of SL signaling [27]. In addition, SMXL7 has been shown to form dynamic nuclear condensates through phase separation. These condensates enrich core SL signaling components, including D14 and MAX2, and mediate transcriptional repression of downstream genes by restricting the binding of specific transcription factors to their target genes. This finding reveals a new mechanism by which nuclear spatial organization regulates SL signaling output [28]. Moreover, plant-specific casein kinases, ARABIDOPSIS EL1-LIKEs (AELs), can phosphorylate SMXL6/7/8, enhance their stability, and reduce MAX2-mediated degradation, thereby attenuating SL signal output and promoting shoot branching [29]. Together, these findings indicate that SL signaling is regulated at multiple levels, and its functional output is coordinately regulated by receptor degradation, nuclear spatial organization, and protein phosphorylation.

In summary, the SL biosynthetic pathway determines the formation of different types of SL molecules, whereas the D14–MAX2–SMXL/D53 module provides a relatively conserved route for SL perception and signal output. In this pathway, SMXL/D53 repressors are degraded, thereby relieving transcriptional repression of SL-responsive genes and potentially contributing to adaptive responses under salt stress.

## 3. Molecular Mechanisms of SLs in Regulating Plant Salt Tolerance

### 3.1. Responses of SL Biosynthesis and Signaling Genes to Salt Stress

Genetic studies indicate that genes involved in SL biosynthesis and signaling function as positive regulators of plant salt stress responses. In Arabidopsis, the salt tolerance of the SL biosynthesis-deficient mutants *max3* and *max4* was significantly reduced, and plant growth was markedly inhibited. The stress-sensitive phenotype of SL-deficient mutants was mainly associated with shoot-related traits, including altered stomatal density, delayed stomatal closure, and accelerated leaf water loss. By contrast, root-related changes were less pronounced. This finding indicates that the regulatory role of SLs in stress responses may be organ-specific [30]. In addition, functional studies of *MAX2* homologs further support the positive role of the SL signaling pathway in salt stress tolerance. In apple (*Malus domestica*), salt stress significantly induced the expression of *MdMAX2*. Functional analysis showed that overexpression of *MdMAX2* could modulate light-responsive developmental processes and enhance the salt stress tolerance of transgenic plants [31]. Similarly, heterologous overexpression of *SsMAX2*, *CsMAX2*, and *GmMAX2a* in *Arabidopsis* also enhanced salt-tolerance phenotypes. Compared with wild-type plants, these overexpression lines exhibited higher antioxidant enzyme activities, greater accumulation of osmotic solutes, and significantly upregulated expression of abscisic acid (ABA) biosynthesis-related genes and multiple stress-responsive genes [32,33,34]. These results suggest that *MAX2* may act as a key regulatory hub that connects SL signaling with developmental regulation and stress-response networks.

Accumulating studies indicate that salt stress can affect the expression of genes involved in SL biosynthesis and signaling, as well as endogenous SL levels. In mycorrhizal *Sesbania cannabina* seedlings, salt stress significantly upregulated the expression of the root SL biosynthesis genes *CCD7* and *CCD8*, as well as the stem signaling gene *MAX2*, and increased endogenous SL levels in seedlings [35]. Similarly, salt stress increased SL accumulation in mycorrhizal lettuce (*Lactuca sativa*), with SL levels rising approximately fivefold under higher salinity. By contrast, salt stress significantly reduced root SL production in non-mycorrhizal lettuce plants [15]. These results suggest that AM symbiosis can influence SL biosynthesis and accumulation in roots under salt stress. Since SLs function as important rhizosphere signaling molecules associated with AMF hyphal branching and symbiosis establishment, changes in their accumulation may be related to the adjustment of plant–rhizosphere interactions and symbiotic adaptation under salt stress. Osmotic stress, as an important component of the early salt stress response, can also affect SL levels in roots. In *Lotus japonicus*, osmotic stress reduced the transcript levels of genes involved in SL biosynthesis and the transporter gene *LjPDR1* in roots, resulting in reduced SL levels in roots and root exudates. Notably, exogenous GR24 treatment suppressed osmotic stress-induced ABA accumulation and reduced the expression of *LjNCED2* in roots, indicating that the decrease in root SLs may be a prerequisite for the normal increase in ABA [36]. Therefore, the response of the SL pathway under salt stress may be associated with mycorrhizal symbiosis and the interaction between SLs and ABA. Genome-wide analyses have further shown that SL pathway-related genes participate in plant responses to salt stress. In grapevine (*Vitis vinifera* L.) and soybean (*Glycine max*), the promoter regions of SL biosynthesis genes (*D27*, *CCD7*, *CCD8*, and *MAX1*) and signaling genes (*D14*, *MAX2*, and *D53*) contain cis-elements responsive to hormones and abiotic stress. These SL-related genes also show tissue-specific expression patterns in roots, stems, and leaves under saline-alkali stress [6,37]. These findings suggest that SL biosynthetic and signaling genes are dynamically regulated components of the salt stress response network, with expression patterns regulated by species, tissue type, and symbiotic status. In summary, SL pathway responses under salt stress may be influenced by AM symbiotic status, ABA dynamics, and tissue-specific regulation. Future studies should integrate time-series transcriptomic analysis, endogenous SL quantification, and root exudate profiling to further clarify the response patterns of the SL pathway under salt stress.

### 3.2. Crosstalk Between SLs and ABA, ROS, and Other Signaling Pathways Under Salt Stress

Plant responses to salt stress involve the coordinated action of multiple hormones and second messengers. Current evidence indicates that SLs exhibit complex crosstalk with early signaling molecules such as ABA, reactive oxygen species (ROS), and calcium ions (Ca^2+^), thereby jointly regulating plant salt tolerance.

ABA is a central regulator of plant responses to salt stress. Both ABA and SLs are derived from carotenoid metabolic pathways, and increasing evidence indicates complex crosstalk between ABA and SLs in plant stress responses. In *Arabidopsis*, treatment with exogenous ABA led to increased expression of the SL biosynthetic genes *MAX3* and *MAX4*. During seed germination and seedling growth, SL-deficient mutants exhibited reduced responsiveness to exogenous ABA compared with the wild type. This suggests that SL signaling may act downstream of ABA or function as a regulatory component of ABA responses [30]. In arbuscular mycorrhizal *Sesbania cannabina*, the application of ABA biosynthesis inhibitors significantly reduced SL levels, whereas H_2_O_2_ scavengers inhibited ABA-induced SL accumulation. These results suggest that ABA may positively regulate SL-mediated salt tolerance through H_2_O_2_ signaling [35]. In addition, genome-wide identification in grape showed that the promoters of genes related to SL biosynthesis and signaling contain ABREs and other hormone- and abiotic stress-responsive cis-acting elements, suggesting that ABA may participate in salt stress responses by regulating the expression of SL-related genes [6]. Studies in apple further revealed the molecular mechanism by which ABA enhances SL signaling. The ABA-responsive factor MdABI5 binds to the *MdMAX2* promoter and activates its transcription. Meanwhile, ABA inhibits the ubiquitin-mediated degradation of MdMAX2 by the E3 ubiquitin ligase MdMIEL1, thereby increasing MAX2 protein stability and enhancing SL signaling [38]. Notably, in dodder-connected tobacco plants, the SL, ROS, and ABA pathways in donor plants negatively regulate dodder-mediated transmission of interplant salt-stress priming signals. Among these pathways, SLs and ROS may regulate systemic signaling through the ABA pathway [39]. These findings indicate that the interaction between SLs and ABA is involved not only in plant stress responses but also in systemic signal transduction between plants. Overall, the functional interaction between SLs and ABA in salt stress responses is mainly reflected in ABA-mediated regulation of SL biosynthesis, SL accumulation, and SL signaling stability, as well as the involvement of SL signaling in ABA-related responses. However, the direct target genes involved in ABA-mediated regulation of the SL pathway and the position of ROS in this signaling hierarchy remain to be clarified.

ROS and Ca^2+^ are key second messengers in the early response to salt stress [40]. Under salt stress, excessive ROS accumulation causes oxidative damage, whereas moderate ROS levels, particularly H_2_O_2_, function as signaling molecules that help activate stress defense responses. In cucumber (*Cucumis sativus*), exogenous GR24 enhanced antioxidant enzyme activity, maintained K^+^/Na^+^ homeostasis, and reduced malondialdehyde (MDA) content by upregulating the expression of key genes involved in oxidative defense and ion homeostasis. The application of an H_2_O_2_ scavenger, a Ca^2+^ channel blocker, or an NADPH oxidase inhibitor significantly suppressed the alleviating effect of GR24. These results indicate that H_2_O_2_ and Ca^2+^ signaling are involved in GR24-mediated regulation of salt tolerance [41]. Further studies have shown that GR24 may modulate H_2_O_2_-related signaling and the expression of *MAPK3/4/6*, thereby alleviating salt stress-induced photosynthetic inhibition and oxidative damage [42]. However, current evidence has not clarified the causal hierarchy among H_2_O_2_ signaling, Ca^2+^ signaling, and MAPK-related pathways in SL-mediated salt tolerance.

In recent years, crosstalk between gaseous signaling molecules and SLs has attracted increasing attention. Hydrogen sulfide (H_2_S) and nitric oxide (NO) participate in tomato salt stress adaptation by modulating SL biosynthesis and signaling. Under salt stress, application of the H_2_S donor NaHS or the NO donor GSNO increased endogenous SL levels in tomato seedlings and upregulated genes involved in SL biosynthesis and signaling. Conversely, the SL biosynthesis inhibitor TIS108 weakened the positive effects of NaHS and GSNO on tomato salt tolerance. Further *SlD27* silencing experiments showed that the role of H_2_S in enhancing salt stress tolerance is associated with endogenous SL biosynthesis [43,44]. These findings suggest that the SL pathway may participate in H_2_S- and NO-mediated salt stress responses. However, the specific position of SLs within this signaling network and the underlying regulatory mechanisms remain to be further elucidated.

SLs contribute to plant salt tolerance by modulating multiple signaling pathways, but the mechanisms underlying these interactions remain unclear. In particular, the early activation of SL signaling under salt stress, as well as the upstream and downstream relationships among different signals, key regulatory factors, and downstream target genes, still need to be further clarified.

### 3.3. Downstream Regulatory Modules of SLs Under Salt Stress

In the canonical SL signaling pathway, SLs mediate the degradation of repressors such as D53/SMXL7 through the receptor D14 and the F-box protein MAX2, thereby relieving repression of downstream signaling. However, SL signaling involves more complex regulatory networks under stress conditions. Under osmotic stress, D14 has been shown to mediate the degradation of the non-canonical substrate SMAX1. This finding suggests that stress-responsive SL signaling is not confined to canonical downstream components and that functional crosstalk may exist between the SL and KAR/KL pathways [45]. In soybean, salt stress delays seed germination and induces the expression of several *GmSMXL* genes, together with upstream components of the KAR/SL signaling pathway. This suggests that SMXL family members may be involved in early salt stress responses through MAX2-mediated KAR/SL signaling [46]. Studies in apple further revealed that the SL signaling repressor MdD53 participates in saline-alkali stress regulation. SL signaling promotes MdD53 degradation, releases its repression of the transcription factor MdbHLH1, and activates the downstream alkaline stress tolerance-related gene *MdAT1*. This regulatory module promotes H_2_O_2_ efflux, reduces oxidative damage, and enhances saline-alkali stress tolerance in apple [47]. This finding broadens our understanding of the role of D53/SMXL repressors in linking SL signaling to stress-responsive transcriptional regulation.

Recent studies suggest that SLs may contribute to salt stress adaptation through epigenetic regulation. Salt stress can reshape DNA methylation at CHG and CG contexts and modulate the expression of stress-responsive genes, thereby supporting adaptive transcriptional reprogramming [48,49]. In tomato, GR24 reduced CHG methylation and activated pathways related to phosphatidylinositol signaling and phenylpropanoid metabolism. These changes were accompanied by increased accumulation of PI signaling-related metabolites, lignin, and phenolic compounds, as well as enhanced activities of phenylpropanoid metabolism-related enzymes. TIS108, an SL biosynthesis inhibitor, weakened the GR24-induced demethylation effect, suggesting that SL-mediated DNA demethylation may represent a potential regulatory mechanism in SL-regulated salt stress responses [50,51].

Overall, SLs participate in salt stress responses through multiple regulatory layers, including canonical and non-canonical signaling, downstream transcriptional regulation, and epigenetic remodeling. However, whether these mechanisms are broadly conserved across plant species and stress conditions remains unclear, and the underlying regulatory framework requires further validation.

## 4. The Role of SLs in Plant Responses to Salt Stress

Salt stress induces multiple physiological and biochemical responses in plants, and SLs contribute to the coordination of these adaptive processes. Current evidence indicates that SL-mediated alleviation of salt stress shows a degree of conservation across different plant species. However, the key target genes and related metabolic pathways involved in SL-mediated salt tolerance remain to be further elucidated.

**Figure 1:**
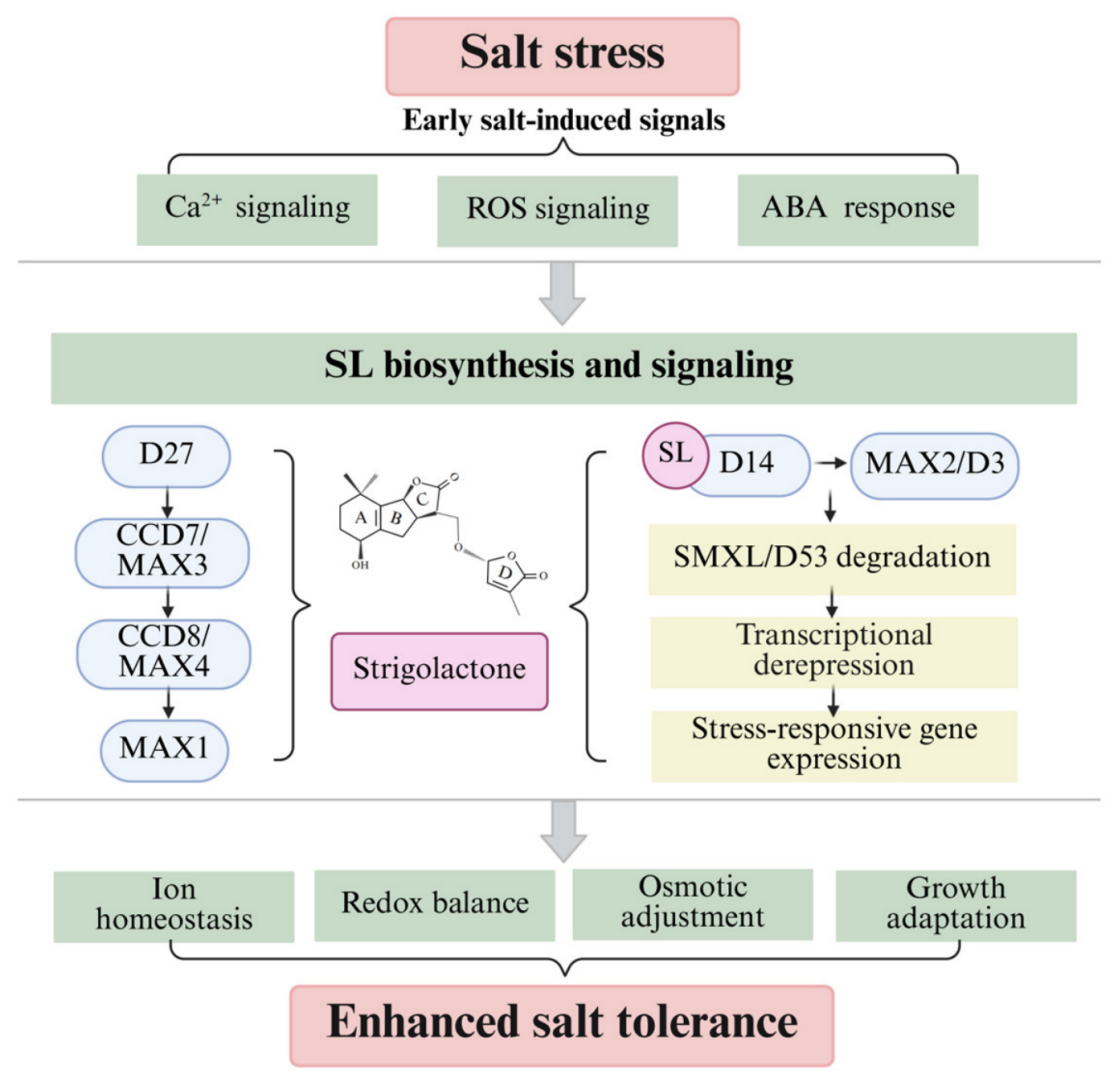
Strigolactone (SL) signaling network linking stress perception to downstream physiological responses and stress adaptation. Salt stress induces Ca^2+^, ROS and ABA signals involved in stress perception. SLs may crosstalk with these pathways and function via the D14–MAX2/D3–SMXL/D53 module to relieve transcriptional repression and regulate stress responses. Gray arrows indicate potential signal flow or regulatory relationships. Created in BioRender. Jieensi, T. (2026) https://BioRender.com/ui3z3cs.

Salt stress rapidly induces ROS accumulation, Ca^2+^ signaling fluctuations, and ABA responses. These early signals are involved in stress perception, signal amplification, and downstream transcriptional regulation [52]. SLs may be associated with ABA, ROS, Ca^2+^, and other signaling pathways and function through the conserved D14–MAX2/D3–SMXL/D53 core module to promote the degradation of transcriptional repressors. This process relieves transcriptional repression and may coordinate multiple physiological processes, including ion homeostasis, redox balance, and cellular osmotic homeostasis, thereby enhancing plant tolerance to salt stress (Figure 1). Although SL-mediated alleviation of salt stress appears to be conserved across diverse plant species, the mechanisms by which early stress signals are connected to the SL perception module and by which SLs further regulate downstream salt tolerance responses remain unclear.

### 4.1. SLs Maintain Ion Homeostasis Under Salt Stress

Salt stress disrupts intracellular Na^+^/K^+^ homeostasis, impairs the normal function of membrane ion transport systems, and causes ionic toxicity and osmotic imbalance, ultimately restricting plant growth and development [53]. Therefore, limiting Na^+^ accumulation while maintaining K^+^ homeostasis is a key physiological basis for plant salt tolerance. SLs participate in the maintenance of ion homeostasis under salt stress by regulating ion uptake and long-distance transport, promoting Na^+^ compartmentalization, and modulating proton pump activity.

Under salt stress, SLs contribute to ionic balance by limiting Na^+^ accumulation, facilitating K^+^ retention or uptake, and thereby preserving a favorable K^+^/Na^+^ ratio. In tomato, *CCD7*-silenced plants are highly sensitive to salt stress. The Na^+^/K^+^ ratio in leaves is about twofold higher than that in the wild type, whereas the contents of K^+^ and Mg^2+^ are significantly reduced. By contrast, ion-related differences between *CCD7*-silenced and wild-type tomato plants were less pronounced in roots, indicating that the regulation of ion homeostasis by SLs may be mainly reflected in the shoots [54]. In addition, exogenous GR24 treatment can significantly reduce Na^+^ accumulation in the leaves of wheat (*Triticum aestivum*), pepper (*Capsicum annuum*), and other crops, decrease electrolyte leakage, improve mineral nutrient uptake, and maintain ion homeostasis [55,56].

At the transcriptional level, SL-mediated changes in ion transporter-related genes provide additional evidence that SLs contribute to the reconstruction of ionic balance under salt stress. In bread wheat, GR24 treatment enhanced the expression of *TaSOS1*, *TaAKT2*, and *TaHAK*, which was accompanied by increased Na^+^ efflux and K^+^ uptake, as well as lower H_2_O_2_ and MDA accumulation [57]. In pepper seedlings, salt stress upregulated *CaSOS1*, *CaSOS2*, and *CaHKT2;2*, but the regulatory effects of exogenous GR24 on these genes differed among genotypes. GR24 also decreased the Na^+^/K^+^ ratio and improved photosynthetic performance and mineral nutrient uptake [58]. In addition, in maize, combined treatment with GR24 and BR modified the expression of K^+^ channel-related genes, such as *AKT2/3* and *KAT2*, and reduced Na^+^ accumulation in roots under salt stress [59].

SLs also enhance the electrochemical driving force for ion transport by stimulating plasma membrane H^+^-ATPase activity. In *Malus hupehensis* seedlings treated with GR24, the AHA family genes (*MhAHA1*, *MhAHA3*, and *MhAHA9*) showed significantly increased expression, together with enhanced H^+^-ATPase activity and organic acid production, thereby alleviating high pH stress [60]. In tomato, SLs also activate H^+^-ATPase activity and facilitate starch accumulation and sucrose degradation, thereby supporting energy supply and osmotic protection under salt stress [61].

### 4.2. SLs Enhance Osmotic Adjustment Under Salt Stress

Salt stress reduces external water potential, leading to cellular water loss and reduced turgor pressure, which seriously affects plant water status and growth [62]. To adapt to this unfavorable environment, plants adjust intracellular osmotic potential by accumulating compatible solutes. Studies have shown that SLs can enhance osmotic adjustment under salt stress.

SLs maintain cellular osmotic balance under salt stress by promoting the accumulation of osmolytes. In crops such as rice, sunflower (*Helianthus annuus*), and ajwain (*Trachyspermum ammi*), GR24 treatment can significantly promote the accumulation of compatible solutes such as proline and glycine betaine, increase leaf osmotic potential and turgor potential, and thereby improve water uptake and osmotic adjustment [14,63,64]. In maize, the combined application of SLs and melatonin further increased the contents of proline, total soluble sugars, and total phenols, while maintaining a high K^+^ level and enhancing osmotic adjustment capacity [65]. In wheat, exogenous GR24 markedly increased the transcript level of *TaP5CS*, a key gene involved in proline biosynthesis, and enhanced proline accumulation [66].

SLs can also promote energy supply and enhance osmotic adjustment by regulating sugar metabolism. In germinating rice seeds, salt stress restricts the conversion of stored reserves by limiting starch degradation and reducing soluble sugar and protein contents. GR24 treatment reversed these changes by increasing amylase activity, including total amylase, α-amylase, and β-amylase, which promoted reserve mobilization and mitigated salt-induced inhibition of early seed growth [67]. In tomato seedlings, GR24 improves osmotic adjustment by activating the *TPS/TPP*-mediated Tre biosynthetic pathway, suppressing *THL*-mediated Tre degradation, and modulating the metabolic balance among Tre, starch, and glucose [68].

### 4.3. SLs Alleviate Oxidative Damage Caused by Salt Stress

Under salt stress, excessive ROS accumulation in plant cells induces oxidative damage, including membrane lipid peroxidation and damage to proteins and nucleic acids [69]. SLs can maintain cellular redox homeostasis and mitigate growth inhibition under salt stress.

SLs contribute to redox homeostasis under salt stress by strengthening antioxidant defense and promoting the AsA–GSH cycle, thereby reducing ROS accumulation, mitigating oxidative damage, and maintaining membrane stability. In rice, cucumber, and maize, GR24 improved the antioxidant capacity of salt-stressed plants by increasing the activities of superoxide dismutase (SOD), peroxidase (POD), catalase (CAT), and ascorbate peroxidase (APX), promoting the accumulation of ascorbic acid (AsA) and glutathione (GSH), reducing the accumulation of ROS and MDA, and inducing antioxidant-related gene expression [14,70,71]. In *Lycium ruthenicum* Murr., GR24 enhanced the activities of key enzymes (APX, GR, DHAR, and MDHAR) in the AsA–GSH cycle, upregulated genes related to starch and sucrose metabolism and GSH synthesis, strengthened antioxidant defense, and significantly improved leaf cell viability and membrane integrity [72]. In *Salvia nemorosa*, GR24 treatment reduced the salt stress-induced elevation of antioxidant enzyme activities, suggesting that SLs may help adjust antioxidant responses after mitigating oxidative damage, thereby maintaining redox homeostasis [73]. In wheat, exogenous GR24 enhanced APX, CAT, and polyphenol oxidase (PPO) activities and decreased MDA, H_2_O_2_, and electrolyte leakage under salt stress. However, guaiacol peroxidase (GPOX) activity varied depending on cultivar and salt stress intensity [66].

### 4.4. SLs Enhance Photosynthetic Efficiency Under Salt Stress

Oxidative stress induced by salt stress can damage cellular and chloroplast structures, lead to stomatal closure, inhibit the activity of photosynthetic enzymes, and significantly reduce the photosynthetic rate and light energy use efficiency [74]. Evidence suggests that SLs can enhance photosynthetic capacity and light energy use efficiency.

SLs alleviate salt stress-induced damage to photosynthetic organs by improving the integrity of chloroplasts and leaf tissue structure. In *Lycium ruthenicum* Murr., GR24 treatment reversed salt-induced chloroplast swelling and deformation and helped maintain the stability of subcellular structures in photosynthetic organs. It also increased the thickness of the leaf epidermis, palisade tissue, and spongy tissue, thereby improving leaf anatomical organization and water retention capacity [72]. Similarly, in rice seedlings, GR24 effectively alleviated chloroplast swelling and thylakoid disintegration caused by salt stress, and maintained chloroplast structural integrity [75]. In cotton (*Gossypium hirsutum*) seedlings, GR24 treatment significantly increased the transcript levels of genes associated with chlorophyll biosynthesis, photosystem I and II components, and photosynthetic electron transport. These results suggest that SLs may contribute to the maintenance of photosynthetic system function through transcriptional regulation of photosynthesis-related genes [76].

SLs may help sustain photosynthetic performance under salt stress by limiting membrane lipid peroxidation, preserving membrane stability, and improving ionic and water balance, thereby reducing stomatal limitation and restricted CO_2_ supply. Evidence from rapeseed (*Brassica napus*), tomato, and rice shows that GR24 enhances antioxidant enzyme activities, lowers MDA accumulation, and promotes osmolyte accumulation, which together mitigate oxidative damage and support cellular homeostasis. These changes are accompanied by increases in net photosynthetic rate (Pn), transpiration rate (Tr), and stomatal conductance (Gs), as well as adjustments in intercellular CO_2_ concentration (Ci), ultimately improving photosynthetic gas exchange under salt stress [77,78,79].

### 4.5. SLs Regulate Root Architecture and Mycorrhizal Symbiosis Under Salt Stress

The root is the primary site where plants perceive and respond to soil salt stress. Salt stress-induced osmotic stress and ion toxicity inhibit root growth and significantly reduce the ability of plants to acquire water and nutrients [80]. As rhizosphere signaling molecules, SLs enhance plant rhizosphere adaptation under salt stress by regulating root development and AMF symbiosis [81].

During seed germination, SLs significantly regulate early root morphogenesis. Under salt stress, radicle length and lateral root number decreased in cucumber. Exogenous GR24 treatment promoted root development, whereas the SL biosynthesis inhibitor TIS108 significantly weakened this alleviating effect, indicating that endogenous SLs contribute to the regulation of early root development [70]. Similarly, GR24 pretreatment significantly alleviated the inhibitory effects of salt stress on seed germination, radicle elongation, lateral root formation, and biomass accumulation in rice and maize during germination [67,71]. At the seedling stage, the regulatory effects of SLs on root architecture were more pronounced, mainly reflected in the promotion of primary root elongation and root hair development. In rice, exogenous GR24 promoted a more developed root system, characterized by greater root length, surface area, and volume, together with enhanced root hair formation and nutrient absorption efficiency [14]. In tomato, GR24 treatment significantly alleviated the inhibitory effects of salt stress on root morphology, increased root activity, and maintained the integrity of internal root anatomical structure [82]. By promoting primary root elongation, root hair formation, and root vitality, SLs provide morphological support for improved plant salt tolerance.

SLs can further enhance plant salt tolerance by mediating the symbiotic interaction between plants and AMF. Salt stress can stimulate root-derived SL secretion. Increased SL secretion promotes AMF colonization and hyphal branching, thereby providing a signaling basis for the establishment of plant–AMF symbiosis. In lettuce, AMF symbiosis significantly improved plant growth, stomatal conductance, and photosystem II efficiency under salt stress, while reducing ABA levels. AMF symbiosis alleviates salt stress by modulating hormonal status and improving photosynthetic performance [15]. Similarly, in AMF-symbiotic *Sesbania cannabina* seedlings, ABA and H_2_O_2_ signals are involved in SL accumulation and SL-induced salt tolerance responses, further suggesting that SL–AMF interactions may cooperate with hormonal and ROS signaling to regulate salt stress adaptation [35].

**Figure 2:**
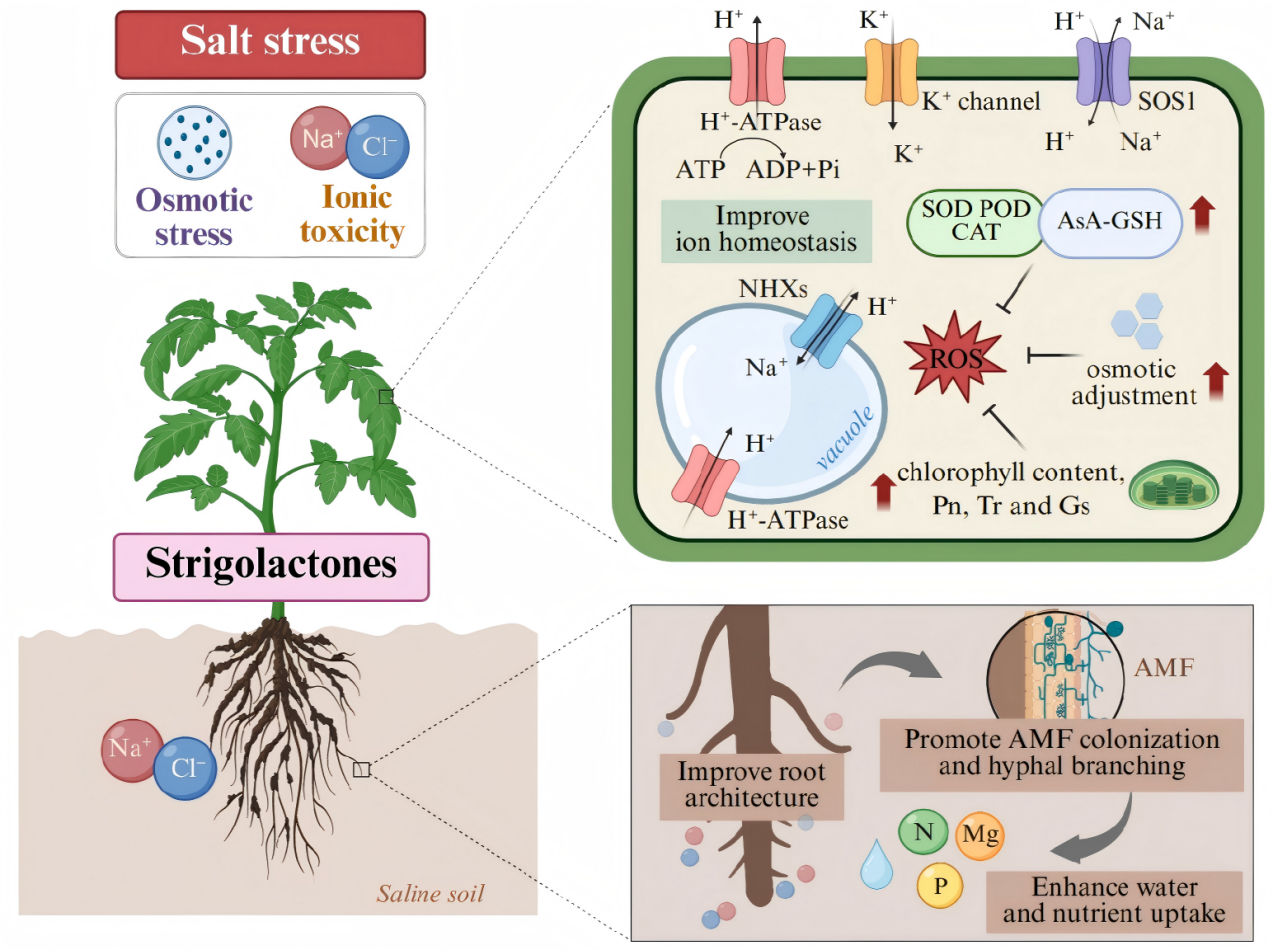
Physiological mechanisms underlying SL-mediated regulation of plant responses to salt stress. Salt stress induces osmotic imbalance and ionic toxicity, leading to disrupted ion homeostasis, redox imbalance, and impaired photosynthetic function. SLs improve Na^+^/K^+^ homeostasis by regulating ion transport systems, including H^+^-ATPase, SOS1, K^+^ channels, and NHXs, which promote Na^+^ efflux, K^+^ uptake, and vacuolar Na^+^ compartmentalization. SLs also strengthen antioxidant defense, restrict excessive ROS accumulation, and alleviate oxidative damage. In addition, SLs promote osmolyte accumulation and help maintain photosynthetic performance. In the rhizosphere, SLs reshape root architecture, facilitate AMF colonization and hyphal branching, and optimize rhizosphere resource acquisition, collectively improving plant adaptation to salt stress. Red arrows indicate enhanced physiological responses, whereas T-bars indicate inhibition or reduction. Created in BioRender. Jieensi, T. (2026) https://BioRender.com/ausihkz.

In summary, SLs coordinate photosynthesis, antioxidant defense, osmotic adjustment, and growth remodeling through integrated molecular, physiological, biochemical, and morphological mechanisms. These coordinated effects highlight the role of SLs in linking multilayered stress responses and shaping salt-tolerant phenotypes, as shown in Figure 2.

## 5. Exogenous Application and Genetic Engineering of SLs

Exogenous application of SLs significantly enhances salt tolerance in different crops, and the main application methods include seed soaking, root application, and foliar spraying. Lower SL doses are often used during seed germination and early seedling establishment, whereas relatively higher doses are more suitable for the middle and later stages of vegetative growth to mitigate salt-induced damage. Representative studies on exogenous GR24 application in different plant species under salt stress are summarized in Table 1, including treatment concentrations, application methods, and major physiological effects. In parallel, genetic regulation of genes involved in SL biosynthesis and signaling provides a potential strategy for enhancing plant salt tolerance. Together, exogenous SL application and SL pathway-based genetic approaches expand the available options for enhancing crop adaptation to saline environments and offer potential routes for improving agricultural productivity in salt-affected soils.

### 5.1. Seed Soaking

Under salt stress, crop seed germination and seedling establishment are highly susceptible to inhibition, which affects early plant establishment and ultimately limits yield formation. Soaking seeds with low concentrations of GR24 can prime regulatory processes related to seed germination and stress adaptation, maintain early physiological homeostasis in seedlings, and provide a foundation for subsequent growth.

In rice, seed soaking with 1.2 μM GR24 mitigated the effects of 80 mM NaCl across different developmental stages. During germination, GR24 promoted seed germination under salt stress mainly by regulating reserve mobilization and endogenous hormone balance [67]. At the seedling stage, GR24 reduced oxidative injury by sustaining photosynthetic pigment levels, activating antioxidant defense, and modulating hormonal homeostasis, thereby strengthening seedling tolerance to salinity [14]. In maize, seed soaking with 0.001–0.1 mg L^−1^ GR24 significantly improved the early establishment of root system morphology in seedlings under 100 mM NaCl stress and enhanced antioxidant defense and osmotic adjustment. Among these treatments, 0.01 mg L^−1^ GR24 showed the strongest alleviating effect, increasing root length by 31.3% and total leaf area by 62% compared with the salt stress control [71]. Similarly, in ajwain, seed priming with 0.001–0.1 mg L^−1^ GR24 before sowing also improved the morphological and physiological status of plants under 100 mM NaCl stress. Among these treatments, 0.001 mg L^−1^ GR24 showed the best effect, significantly increasing shoot fresh weight, enhancing antioxidant defense, and promoting the accumulation of osmotic adjustment substances [64].

Seed soaking treatment is easy to apply and is suitable for improving stress tolerance at the early growth stage. It can induce early stress-responsive physiological changes during seed imbibition, providing a protective basis for seedlings to withstand subsequent salt stress. However, its field application remains limited by challenges such as the high cost of large-scale seed treatment. Further studies are needed to determine the optimal soaking concentration and duration for different crops to avoid adverse effects caused by excessive concentrations. In addition, residual SLs in soil may induce the germination of parasitic weed seeds, potentially increasing field risks.

### 5.2. Root Application

Root application of SLs, including addition to nutrient solutions or rhizosphere soil application, allows SLs to act directly on root tissues, rapidly activate root-associated signaling pathways, strengthen root stress responses, and effectively alleviate salt-induced inhibition of plant growth. This application method provides an important approach for the precise regulation of the rhizosphere.

In filter-paper germination assays, 1–20 μM GR24 mitigated the suppressive effect of 50 mM NaCl on cucumber seed germination. GR24 enhanced antioxidant capacity by increasing antioxidant enzyme activities and upregulating related genes. It also promoted radicle elongation and lateral root formation. Among these treatments, 10 μM GR24 showed the strongest alleviating effect [70]. In pot experiments, the application of 0.18 μM GR24 significantly improved shoot and root growth in rapeseed, and markedly alleviated the damage caused by 100 and 200 mM NaCl stress by regulating tryptophan metabolism, plant hormone signal transduction, and the expression of photosynthesis-related genes [77]. Under 180 mM NaCl stress, the application of 1.65 nM BR and 1 μM GR24, either alone or in combination, alleviated salt injury in maize. Among these treatments, the combined treatment was more effective, as it significantly promoted seedling growth, increased antioxidant enzyme activity, and reduced root Na^+^ accumulation [59]. In addition, hydroponic experiments showed that 15 μM GR24 contributed to salt stress tolerance in tomato seedlings exposed to 150 mM NaCl by modulating trehalose biosynthetic processes [68].

Root application can act directly in the rhizosphere and rapidly activate the salt tolerance signaling network. However, field application still faces several challenges, including the uneven distribution of SL analogues in soil, fluctuations in their effective concentrations in the rhizosphere, and the high cost of large-scale use in crop production. Moreover, where parasitic plant seed banks are present in field soils, SL application may increase the risk of stimulating parasitic weed germination. Future strategies should focus on optimizing application concentrations, improving targeted rhizosphere delivery, and integrating agronomic measures such as crop rotation or suicidal germination.

### 5.3. Foliar Spraying

Foliar application of SLs is a convenient and effective strategy for salt stress mitigation. SLs can be rapidly absorbed by leaves, thereby activating leaf antioxidant defense, enhancing photosynthetic protection, and improving plant adaptation to salt stress.

At the seedling stage of rice, foliar spraying with 0.5 mg L^−1^ GR24 significantly alleviated salt stress-induced damage to the photosynthetic system, effectively reduced ROS accumulation, and reduced salt stress-induced growth inhibition [75]. In a pepper pot experiment, soil salinity was maintained at 6.13 dS m^−1^, and 10–30 μM GR24 was sprayed onto the leaves to evaluate its salt-alleviating effect. Among these treatments, 20 μM GR24 showed the best effect, significantly increasing plant height, dry weight, chlorophyll content, and fruit yield [56]. Similarly, in a pepper hydroponic experiment, 20 μM GR24 alleviated the effects of 100 mM NaCl stress and promoted the responses of genes related to ion homeostasis and SL signaling [58]. Under salinity levels of 5 and 10 dS m^−1^, spraying 10 μM GR24 combined with PGPR inoculation at the pollination stage of wheat synergistically enhanced antioxidant defense, maintained ion homeostasis, and improved physiological traits and yield. The combined treatment showed stronger salt-alleviating effects than either GR24 or PGPR treatment alone [55].

Compared with seed soaking and rhizosphere application, foliar spraying is simple and effective. It can rapidly regulate stomatal changes and antioxidant responses and has potential for large-scale field application. However, this method is easily affected by light, rainfall, and high temperature, and its application cost remains high. In the future, the efficient and large-scale use of SL foliar spraying in saline-alkali agriculture should be promoted by improving the stability of foliar spray formulations and clarifying the optimal spraying concentrations for different crops and growth stages.

### 5.4. Genetic Engineering Strategies

In addition to exogenous SL application, genetic manipulation of key components in the SL pathway offers a promising approach for improving crop salt tolerance. Representative genetic and functional evidence for SL biosynthesis- and signaling-related genes in plant salt stress tolerance is summarized in Table 2. Accumulating evidence suggests that genes involved in SL biosynthesis and signaling not only respond to salt stress but also participate in the regulatory networks that shape salt-tolerance traits.

Mutants defective in SL biosynthesis usually exhibit stronger growth inhibition and higher stress sensitivity under salt stress, whereas exogenous GR24 can partially alleviate salt-induced phenotypes, indicating that endogenous SLs positively regulate plant salt tolerance [30]. In tomato, H_2_S can increase endogenous SL levels by inducing *SlD27* expression, while silencing *SlD27* largely abolishes the H_2_S-mediated enhancement of salt tolerance [43]. Similarly, tomato plants silenced for *CCD7*, a key SL biosynthesis gene, show greater sensitivity to salt stress, accompanied by reduced proline, K^+^, and Mg^2+^ accumulation and increased MDA content. These findings suggest that key genes in the SL biosynthesis pathway may serve as important candidate targets for the genetic improvement of salt tolerance [54].

In addition, the key gene *MAX2* in SL signaling has also shown high application potential. Salt stress induces *SsMAX2* expression, and overexpression of *SsMAX2* enhances the survival of Arabidopsis under salt stress, alleviates chlorophyll degradation, and modulates ion homeostasis-related gene expression [32]. Similarly, heterologous overexpression of soybean *GmMAX2a* in Arabidopsis promotes root growth and fresh weight accumulation under salt stress, while significantly inducing the expression of stress-responsive and ion homeostasis-related genes [34].

Genes involved in SL biosynthesis and signaling represent promising targets for improving crop salt tolerance. Their precise manipulation through gene editing or overexpression may offer useful strategies for salt-tolerance breeding. However, SLs regulate both stress responses and plant architecture. For example, heterologous overexpression of apple *MdD14* in *Arabidopsis thaliana* increased fresh weight, primary root length, and lateral root number under salt stress, whereas it suppressed shoot branching under normal conditions and enhanced sensitivity to GR24-induced inhibition of hypocotyl elongation. This indicates that strengthening SL signaling may influence stress adaptation and developmental regulation simultaneously [83]. Therefore, the breeding application of SL-related genes requires an integrated assessment of salt tolerance, growth, developmental traits, and yield-related performance. Stress-inducible or tissue-specific expression may be useful for balancing stress tolerance with normal plant growth. Future studies should further validate these candidate genes in target crops through stable genetic transformation, genome editing, and field trials under saline-alkali conditions, thereby clarifying their practical value in salt-tolerance breeding.

**Figure 3:**
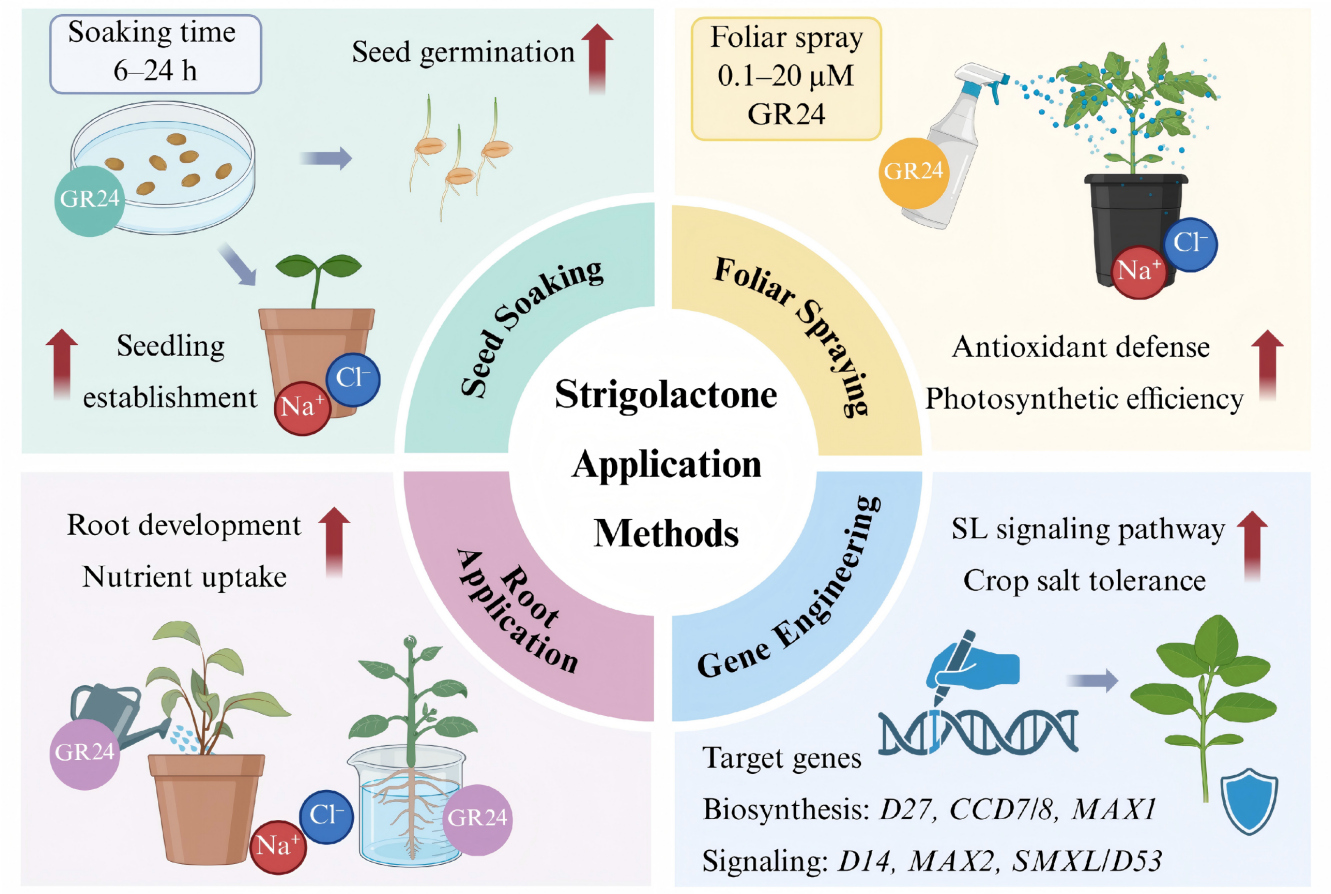
Application methods of strigolactones (SLs) for improving plant salt tolerance. SL application strategies mainly include seed soaking, foliar spraying, root application, and gene engineering. Seed soaking is commonly used during seed germination and seedling establishment to promote early growth under salt stress. Foliar spraying enhances antioxidant defense and photosynthetic efficiency, whereas root application contributes to root development and nutrient uptake. Gene engineering may improve crop salt tolerance by targeting genes involved in SL biosynthesis and signaling. Red arrows indicate enhanced salt-tolerance-related responses after SL application. Created in BioRender. Jieensi, T. (2026) https://BioRender.com/mr01rzd.

In conclusion, exogenous SL application and genetic regulation of key genes in the SL pathway represent two important strategies for improving crop salt tolerance, as shown in Figure 3.

## 6. Conclusions and Future Perspectives

Genetic and physiological studies collectively indicate that SLs contribute positively to plant salt tolerance. Under salt stress, SL biosynthesis and signaling may be modulated through crosstalk with other signaling pathways. SLs effectively alleviate salt-induced oxidative stress, ionic toxicity, and osmotic imbalance, while maintaining PSII efficiency and carbon fixation capacity. In addition, by regulating root architecture and mycorrhizal symbiosis, SLs help coordinate plant growth with stress adaptation.

SLs show considerable potential for agricultural application. Exogenous application has the advantages of operational simplicity and rapid physiological response. The effective concentration of GR24 is usually within the range of 0.1–20 μM, whereas excessive concentrations may inhibit plant growth. However, plant sensitivity to GR24 varies substantially depending on species, developmental stage, organ type, and environmental conditions. Therefore, practical application requires crop-specific optimization of SL dosage according to actual growth conditions. Currently, the stability and cost of synthetic SL analogues remain major constraints on their field-scale application, and most available evidence is still derived from laboratory or controlled-environment studies. In addition to exogenous application, regulation of key genes involved in SL biosynthesis and signaling also provides an important strategy for the genetic improvement of salt tolerance. Biosynthesis genes such as *D27*, *CCD7*, *CCD8*, and *MAX1* regulate endogenous SL levels, whereas signaling components such as *D14*, *MAX2*, and *SMXL/D53* participate in SL signal output and downstream stress-response regulation. Therefore, these genes may serve as candidate targets for overexpression, gene editing, or expression modulation. However, constitutive or excessive regulation of SL biosynthesis and signaling output may affect plant architecture, biomass accumulation, and yield formation. Future SL pathway-based strategies for improving salt tolerance should consider expression intensity and tissue- or organ-specific regulation to coordinate salt stress adaptation with crop productivity.

SLs are important signaling molecules involved in plant growth, development, and abiotic stress responses. However, the molecular mechanisms by which SLs regulate salt stress responses remain incompletely understood. Although D53/SMXL degradation is known to relieve transcriptional repression, it remains unclear which stress-responsive transcription factors are regulated downstream of this process and how they contribute to salt-specific responses. In addition, systematic evidence for SL-mediated epigenetic regulation is still lacking. At present, many studies on SL-mediated salt stress responses still rely on exogenous GR24 treatment, with emphasis on morphological, physiological, and biochemical changes. However, GR24-induced effects cannot be regarded as fully equivalent to the functions of endogenous SLs, because rac-GR24 may activate both D14-mediated SL signaling and KAI2-mediated KAR/KL-like signaling. Therefore, future studies should integrate endogenous SL quantification with genetic validation to define the roles of SLs in salt stress responses more precisely.

Future studies should further elucidate the crosstalk between SLs and ABA, ROS, Ca^2+^, and other signaling pathways during salt stress responses and validate the hierarchical relationships among these signaling components to clarify the SL-mediated signaling network under salt stress. At the same time, more attention should be given to the interaction mechanisms between SLs and rhizosphere microorganisms, mycorrhizal symbiosis, and the soil nutrient environment. It is also necessary to promote the development of exogenous SL formulations, precise application strategies, and field validation of gene-edited materials, so as to enhance their application potential in salt-tolerant cultivation and stress-resistant breeding.

Overall, SLs function as important signaling molecules that connect plant growth, stress responses, and rhizosphere ecological interactions, thereby acting as integrative regulators of plant salt tolerance. As understanding of SL biosynthesis, signaling, and application mechanisms continues to advance, SLs are expected to provide new strategies for saline-alkali land utilization, salt-tolerant crop improvement, and stress-resilient agricultural management.

## Figures and Tables

**Table 1:** Application of exogenous GR24 in different plant species under salt stress.

Crop	Salt Stress	GR24 Treatment	GR24 Application Method	Effect	Reference
Tomato (*Solanum lycopersicum* L.)	Four-leaf seedlings treated with NaCl (150 mM)	15 μM	Nutrient solution	Enhanced chlorophyll, carotenoid, and flavonoid biosynthesis; upregulated phosphoinositide signaling genes; activated phosphoinositide signaling	[50]
	Four-leaf seedlings treated with NaCl (150 mM)	15 μM	Nutrient solution	Reduced L-phenylalanine and cinnamic acid contents; upregulated phenylpropanoid metabolism genes; increased PAL, CYP98A3 and BGLU activities	[51]
	10-day-old seedlings treated with NaCl (100 mM) for 48 h	4 μM	Petri dish treatment	Improved K^+^ content, H^+^-ATPase activity; reduced oxidative stress; enhanced starch accumulation	[61]
	Four-leaf seedlings treated with NaCl (150 mM)	15 μM	Nutrient solution	Improved biomass accumulation, chlorophyll index and photosynthetic parameters; enhanced proline and protein contents	[43]
	Four-leaf seedlings treated with NaCl (150 mM)	15 μM	Nutrient solution	Increased root length, root fresh and dry weight, and root/shoot ratio; enhanced root activity	[82]
	Seedlings at 25 DAS treated with NaCl (150 mM)	2 μM	Foliar spray	Improved stem diameter, biomass accumulation, and leaf area; increased photosynthetic parameters	[78]
	Four-leaf seedlings treated with NaCl (150 mM)	15 μM	Nutrient solution	Improved root morphological parameters and root activity; increased endogenous SL content; upregulated SL biosynthesis and signal transduction genes	[68]
Rice (*Oryza sativa* L.)	Seeds treated with NaCl (80 mM) during germination	1.2 μM	Seed soaking	Improved germination rate, radicle, and plumule length; enhanced SOD, CAT, and APX activities; reduced MDA; increased SLs, IAA, GA3, and CTK	[14]
	Three-leaf seedlings treated with NaCl stress at 0.4% (*w*/*w*) of soil weight	0.5 mg L^−1^	Foliar spray	Enhanced photosynthetic performance and chlorophyll fluorescence; stabilized chloroplast structure; reduced ROS, MDA, and Na^+^/K^+^ ratio	[75]
	Seedlings treated with NaCl (200 mM) for 48 h	0.1, 0.2, 1, 5 μM (optimal 1 μM)	Nutrient solution	Enhanced chlorophyll content and photosynthetic parameters; reduced MDA; increased POD and SOD activities	[79]
Wheat (*Triticum aestivum* L.)	Seedlings treated with NaCl (5 and 15 dS m^−1^) from 3–4 leaf stage to tillering	10 μM	Foliar spray	Improved grain yield, harvest index, and RWC; reduced EL and MDA; enhanced APX and POX activities	[66]
	One-month-old seedlings treated with NaCl (5 and 10 dS m^−1^)	10 μM	Foliar spray	Enhanced APX, CAT, PPO activities; increased K^+^, chlorophyll, and carotenoid contents; reduced MDA and H_2_O_2_; modulated ion channel and stress-related genes	[57]
	Plants at the beginning of the stem elongation stage treated with NaCl (5 and 10 dS m^−1^)	10 μM	Foliar spray	Improved growth, photosynthetic pigments, and carbohydrate metabolism; reduced Na^+^ accumulation	[55]
Maize (*Zea mays* L.)	One-week-old seedlings treated with NaCl (100 mM) for 15 days	25 μM	Foliar spray	Enhanced SOD, POD, and CAT activities; reduced MDA and Na^+^; increased proline, soluble protein, and total phenolic	[65]
	Seedlings treated with NaCl (180 mM)	1 μM	Irrigation water	Regulated carbon fixation, TCA cycle, starch and sucrose metabolism, hormone signaling, and ion transport-related genes	[59]
	Seedlings treated with NaCl (100 mM)	0.001, 0.01, 0.1 mg L^−1^ (optimal 0.01 mg L^−1^)	Seed soaking	Enhanced SOD, POD, CAT, ascorbic acid, proline, and glycine betaine; reduced H_2_O_2_ and MDA	[71]
Cucumber (*Cucumis sativus* L.)	Seeds treated with NaCl (50 mM) during germination	1, 5, 10, 20 μM (optimal 10 μM)	Petri dish treatment	Improved germination rate, radicle length, and lateral root number; reduced H_2_O_2_	[70]
	Two-week-old seedlings treated with NaCl (150 mM)	1.0 μM	Foliar spray	Increased CAT, SOD, POD, and APX activities; elevated the expression of antioxidant enzyme-related genes, NADPH oxidase-related genes, *CDPKs*, *SOS1*, *CIPK2*, and *CBL3*	[41]
Pepper (*Capsicum annuum* L.)	Seedlings treated with NaCl (100 mM)	10, 20 μM (optimal 20 μM)	Foliar spray	Regulated genes associated with Ca^2+^ signaling, SL biosynthesis/signaling, photosynthesis, aquaporin transport, and ion homeostasis	[58]
Chili Pepper (*Capsicum annuum* L.)	Seedlings treated with salinity stress (EC 6.13 dS m^−1^)	10, 20, 30 µM (optimal 20 µM)	Foliar spray	Improved plant height, dry weight, branches, fruit yield, chlorophyll content, N/P/K uptake	[56]
Apple (*Malus hupehensis* Rehd.)	Four-leaf seedlings treated with salinity-alkalinity stress (100 mM NaCl + 100 mM NaHCO_3_, 1:1)	10, 100, 1000 µM (optimal 100 µM)	Nutrient solution	Increased chlorophyll content and organic acid production; upregulated Na^+^/K^+^ transporter and H^+^-ATPase genes	[60]
*Lycium ruthenicum* Murr.	Three-leaf seedlings treated with NaCl (250 mM)	1, 5, 10 µM (optimal 5 µM)	Foliar spray	Improved stomatal aperture, chloroplast ultrastructure, and AsA-GSH cycle; upregulated stress-related genes	[72]
Cotton (*Gossypium hirsutum* L.)	Seedlings treated with NaCl (200 mM)	2.5, 5, 10, 15 µM (optimal 10 µM)	Foliar spray	Reduced ROS, MDA, and H_2_O_2_; upregulated antioxidant enzyme, chlorophyll biosynthesis and photosynthesis genes	[76]
Ajwain (*Trachyspermum ammi* L.)	77-day-old seedlings treated with NaCl (100 mM)	0.001, 0.01, 0.1 mg L^−1^ (optimal 0.001 mg L^−1^)	Seed priming (3 h)	Increased shoot length, fresh weight, SOD, POD, and CAT activities, proline and GB content; reduced MDA	[64]
Ornamental sunflower (*Helianthus annuus* cv. Vincent’s Choice)	Plants treated with NaCl (150 mM)	0.001, 0.01, 0.1 mg L^−1^ (optimal 0.01 mg L^−1^)	Foliar spray	Increased proline, glycine betaine, SOD, CAT, and POD activities; modulated Na^+^, K^+^, and Ca^2+^ accumulation.	[63]
Salvia (*Salvia nemorosa* L.)	Plants treated with NaCl (100, 200 and 300 mM)	0, 0.1, 0.2, 0.3, 0.4 µM (optimal 0.3 µM)	Foliar spray	Improved Fv/Fm, gas exchange, and essential oil yield; reduced MDA and H_2_O_2_; modulated antioxidant enzyme and glutathione-related responses.	[73]
Rapeseed (*Brassica napus* L.)	Seedlings treated with NaCl (100 and 200 mM)	0.18 µM	Irrigation water	Improved chlorophyll content and photosynthetic rate; reduced MDA; regulated genes in hormone signal transduction and photosynthesis	[77]

**Table 2:** Genetic and functional evidence for SL-related genes and mutants in plant salt stress tolerance.

Species	Mutant/Genetic Material	Key Findings	Reference
*Arabidopsis thaliana*	d14-1 mutant	D14 can mediate SMAX1 degradation, indicating crosstalk between SL and KAR/KL signaling; SMAX1 degradation contributes to osmotic stress adaptation.	[45]
*Sapium sebiferum*	*SsMAX2* overexpression in Arabidopsis	*SsMAX2* overexpression enhances salt, drought, and osmotic stress tolerance, mainly through antioxidant defense, osmotic adjustment, and ABA-related gene regulation.	[32]
Soybean (*Glycine max*)	*GmMAX2a* overexpression in Arabidopsis	*GmMAX2a* is induced by salt, alkali, and drought stresses. Its overexpression improves stress tolerance and is associated with activation of stress-responsive genes.	[34]
Cucumber (*Cucumis sativus*)	*CsMAX2* overexpression in Arabidopsis	*CsMAX2* positively regulates salt, drought, and ABA stress tolerance, possibly through ABA signaling and activation of stress-responsive genes.	[33]
Apple (*Malus domestica*)	*MdMAX2* overexpression in Arabidopsis	*MdMAX2* promotes anthocyanin accumulation, photomorphogenesis, and salt/drought stress tolerance.	[31]
Apple (*Malus domestica*)	*MdMAX2*	The MdMIEL1–MdABI5–MdMAX2 module links ABA and SL signaling; ABA regulates *MAX2* at both transcriptional and protein-stability levels.	[38]
Tomato (*Solanum lycopersicum*)	*SlCCD7*-silenced plants	Endogenous SL deficiency increases salt sensitivity; SLs enhance salt tolerance by regulating ion accumulation, osmotic adjustment, and oxidative stress responses.	[54]
Tomato (*Solanum lycopersicum*)	*SlD27*-silenced plants	H_2_S enhanced salt tolerance by upregulating the SL biosynthesis-related gene *SlD27*; *SlD27* silencing reduced endogenous SL levels and largely abolished NaHS-mediated salt stress alleviation.	[43]

## Data Availability

No new data were created or analyzed in this study.
